# Genetic Diversity and Phylogenetic Analysis of Five Cultrinae Species in West Dongting Lake Based on *Cytb* and *COI* Genes

**DOI:** 10.3390/biology14121802

**Published:** 2025-12-18

**Authors:** Yihan Ma, Jia Pan, Haiqi Li, Peng Liu, Weikun Zeng, Boyong Peng, Bin Wang, Zhongyuan Shen, Xiaoyang Mo

**Affiliations:** 1 Vertebrate Zoology Laboratory, College of Life Science, Hunan Normal University, Changsha 410081, China; 2 Yuelushan Laboratory Aquatic Variety Breeding Centre, Hunan Normal University, Changsha 410081, China; 3 Hunan West Dongting Lake National Nature Reserve, Hanshou 415900, China; 4 College of Biological and Food Engineering, Huaihua University, Huaihua 418000, China

**Keywords:** genetic diversity, phylogeny, Cultrinae, Western Dongting Lake

## Abstract

Aiming to understand the genetic diversity of *Culter* and *Cultrichthys* fishes in West Dongting Lake following the implementation of fishing ban, this study analyzed the genetic diversity and phylogeny of five Cultrinae species based on mitochondrial *Cytb* and *COI* gene sequences. For *Cytb*, 50 haplotypes were identified with high overall genetic diversity (*H_d_* = 0.954, *π* = 0.04765, *K* = 53.506). Similarly, *COI* yielded 48 haplotypes (*H_d_* = 0.950, *π* = 0.03251, *K* = 36.414). Interspecific comparisons revealed that *Culter mongolicus* and *Culter alburnus* exhibited the highest genetic diversity, while *Cultrichthys erythropterus* showed the lowest. Demographic inferences from neutrality tests and mismatch distribution analyses differed between markers. Based on *Cytb*, populations of *C. erythropterus* and *C. oxycephaloides* appeared to be at neutrality, whereas *C. mongolicus*, *C. alburnus*, and *C. dabryi* significantly deviated, suggesting historical expansion. Mismatch distributions supported recent expansion in *C. mongolicus* and *C. dabryi.* In contrast, *COI*-based neutrality tests indicated expansion in *C. mongolicus*, *C. alburnus*, and *C. dabryi*, with mismatch results confirming this signal for *C. dabryi*. The negative values of *Tajima’s D* and *Fu’s Fs* observed across all five species reflect an accumulation of low-frequency alleles within populations, which may indicate a recent rapid demographic recovery. These findings provide genetic evidence that the genetic diversity of these Cultrinae species has likely been recovering since the fishing ban took effect on 1 January 2020. Further conservation strategies are therefore recommended to promote population recovery and enhance genetic diversity.

## Simple Summary

Genetic diversity and phylogenetic analyses offer valuable insights into species status. In this study, we examined the genetic diversity and phylogenetic relationships of five Cultrinae species in West Dongting Lake using *Cytb* and *COI* gene markers after the implementation of a ten-year fishing ban. Our aim was to enhance the understanding of genetic diversity within the genera *Culter* and *Cultrichthys*. The findings provide preliminary yet encouraging genetic evidence that the fishing ban may be contributing positively to the recovery of key fish populations in the region. This study establishes an important baseline for future monitoring and underscores the value of evidence-based conservation measures.

## 1. Introduction

Mitochondrial DNA serves as a crucial molecular marker in the domains of population genetics and phylogenetic studies [1,2,3]. This significance arises from its maternal inheritance pattern, lack of recombination, relatively short sequence length, and rapid evolutionary rate [4,5]. In recent years, there has been ongoing advancement and extensive application of DNA sequencing technologies and bioinformatics in the identification of fish species, investigation of phylogeny, exploration of population genetics, progressive hybridization processes, and ecogeography [6,7]. Among the genes under examination, Cytochrome b (*Cytb*) and Cytochrome c Oxidase subunit I (*COI*) are regarded as particularly suitable for studying genetic diversity in fish populations [8,9]. This suitability is attributed to the well-defined structure and function of these genes alongside their moderate evolutionary rates.

Prolonged anthropogenic pressures, including overfishing, hydraulic engineering projects, lake reclamation, and water pollution, have led to a steady decline in migratory fish species within Yangtze River catches [10,11,12]. Concomitantly, the fishery harvest has exhibited a pronounced shift toward miniaturization and younger age structures [13]. To address this pressing issue, the Ministry of Agriculture and Rural Affairs of the people’s Republic of China issued the “Notice on the Scope and Duration of Fishing Ban in Key Water Areas of the Yangtze River Basin” in 2019. The notice mandates a comprehensive ten-year, year-round fishing prohibition in the main stem and major tributaries of the Yangtze River, effective from 1 January 2021. This policy represents a pivotal step toward ecosystem recovery through the implementation of an extensive decade-long fishing moratorium across the entire Yangtze River basin. Culterinae species are widely distributed across China and represent an economically important fish group in inland waters, where they constitute a significant portion of fishery catches in many lakes and reservoirs. Previous studies on three Culterinae species (*Culter alburnus*, *Culter mongolicus*, and *Culter dabryi*), based on *Cytb* and *COI* gene analyses of specimens collected from 2019 to 2020, revealed low genetic diversity and a trend toward genetic homogenization within these populations [14,15]. The implementation of the ten-year fishing ban policy is likely to result in new adjustments to the genetic distribution pattern.

Western Dongting Lake (111°57′–112°17′ E, 28°47′–29°07′ N) serves as the southwestern outlet of Dongting Lake, receiving inflows from the Yuan and Li Rivers while maintaining hydrological connectivity with the Songzi and Taiping waterways. Species of the genera *Culter* and *Cultrichthys*, classified within the subfamily Cultrinae (Cyprinidae) [16], constitute an economically significant fishery in this region. Previous ichthyological surveys in Western Dongting Lake have largely focused on conventional resource assessments, with comparatively limited attention directed toward the genetic diversity and phylogeny of naturally distributed Cultrinae populations in the lacustrine environment. Therefore, assessing the genetic diversity of dominant Cultrinae species during the initial phase of the fishing ban holds practical importance for evaluating, conserving, and utilizing their germplasm resources. In this study, mitochondrial *Cytb* and *COI* gene sequences were determined and combined for five Cultrinae species to analyze the genetic structure, population evolutionary history, and phylogenetic relationships of these taxa. The aim is to elucidate the distribution patterns and population dynamics of genetic diversity in Cultrinae within Western Dongting Lake, thereby providing a scientific basis for the conservation and sustainable utilization of these species.

## 2. Materials and Methods

### 2.1. Sample Collection and Processing

The present study was commissioned by the scientific research of Hunan Western Dongting Lake National Nature Reserve (from July to September 2022, and the utilisation of fishing gears and vessels was approved and agreed by the Reserve, the Law Enforcement Brigade and the relevant departments. A range of conventional fish monitoring techniques were utilised in the course of conducting fish resource surveys, including trawling, gillnetting and ground cages. The identification of fish species was based on the Record of Fishes of Hunan Province [17]. A total of 122 individuals from the genera *Culter* and *Cultrichthys* were collected, comprising 32 specimens of *Culter alburnus*, 32 specimens of *Culter mongolicus*, 39 specimens of *Culter dabryi*, 4 specimens of *Culter oxycephaloides*, and 15 specimens of *Cultrichthys erythropterus*. Following the measurement of each fish’s length and weight, the pectoral fins were excised and placed into sterile, enzyme-free centrifuge tubes (1.5 mL). The fins were preserved in 95% ethanol, assigned a unique identification number, and stored in a refrigerator maintained at −20 °C. The animal study protocol was approved by the Institutional Review Board (or Ethics Committee) of ethics committee of Hunan Normal University.

### 2.2. DNA Extraction, PCR Amplification and Sequencing

The genomic DNA was extracted using the TIANamp Genomic DNA Kit (Model DP304, Tiangen Biotech (Beijing) Co., Ltd., Beijing, China). The primers for the amplification of the *Cytb* gene were L14724 (GACTTGAAAAACCACCGTTG) and H15915 (CTCCGATCTCCGGATTACAAGAC) [18]; the primers for the amplification of the *COI* gene were LCOIa (CCTACCTGTGGCAATCACRCGC) and HCOI (GTGAATAGGGGGAATCAGTG) [19].

Reaction system (50 μL): The following components are essential for the successful execution of the PCR: a DNA template of 22 μL, two primers at 2 μL each (one upstream and one downstream), 25 μL of 2× SanTaq PCR Master Mix (which contains blue dye), and 19 μL of ddH_2_O. The procedure for the polymerase chain reaction (PCR) is as follows: initial denaturation at 94 °C for 4 min, followed by denaturation at 94 °C for 45 s, annealing at 56 °C for 40 s, and extension at 72 °C for 1 min, repeated over a total of 35 cycles. A final extension step was performed at 72 °C for an additional duration of 10 min, after which the samples were stored at a temperature of 4 °C. The amplification products were analyzed using agarose gel electrophoresis with a concentration of 1% and subsequently sent to Sangon BiotechCo., Ltd. (Shanghai, China) for sequencing.

### 2.3. Data Analysis

Sequence comparison was conducted using BioEdit 7.0 [20]. Subsequently, the base composition, conserved sites, variant sites, parsimony informative sites, and self-degenerate sites of each gene’s sequences were analyzed using MEGA v6.0 [21]. Intra- and interspecific genetic distances were calculated employing the Kimura two-parameter model. Haplotype analysis was performed utilizing DnaSP v5.10 software [22], which facilitated the calculation of various genetic parameters including haplotype diversity (*H_d_*), nucleotide diversity (*π*), and the mean number of nucleotide differences (*K*). Haplotype network diagrams were constructed in PopART based on the Median Joining Network method [23]. Neutrality tests and mismatch distribution analyses were executed on the haplotypes using Arlequin 3.5.1.2 [24], yielding results for *Tajima’s D* and *Fu’s Fs* neutrality tests as well as mismatch distribution maps.

The optimal nucleotide substitution models were selected based on the Bayesian Information Criterion (BIC) using jModelTest, with GTR applied to *Cytb* and HKY to *COI*. The Maximum Likelihood (ML) tree was constructed utilizing RAxML-NG [25], while the Bayesian Inference (BI) tree was generated using MrBayes 3.2 [26]. In this study, *Megalobrama skolkovii* and *Parabramis pekinensis* served as outgroups, with sequence identifiers for M*egalobrama skolkovii* (*Cytb*: AF051871; *COI*: NC_024422.1) and *Parabramis pekinensis* (*Cytb*: AF051874; *COI*: NC_022678.1), respectively. The confidence of branch nodes was evaluated through Bootstrap values (BP) and Posterior probabilities (PP).

## 3. Results

### 3.1. Gene Sequence Characteristics

A total of 122 sequences for both the *Cytb* and *COI* genes were obtained. Following alignment and trimming of low-quality terminal bases, homologous conserved sequences of 1123 bp for *Cytb* and 1135 bp for *COI* were retained for subsequent analysis. The base composition of each gene sequence is presented in Table 1.

For the *Cytb* gene, the alignment of 1123 bp comprised 939 conserved sites, 184 variable sites, 165 parsimony-informative sites, and 19 singleton sites. The average nucleotide composition was as follows: T: 27.34%, C: 29.04%, A: 29.11%, and G: 14.52%. This indicates a pronounced anti-G bias. The average content of T + A was calculated to be 56.45%, while the average content of G + C was found to be 43.55%. In contrast, the *COI* gene sequence contained a total of 987 conserved sites, along with 144 variant sites,132 parsimony-informative sites, and12 singleton sites. An anti-G bias was also evident in this gene; mean nucleotide frequencies were determined as T:28.89%, C:26.31%, A:26.18%, and G:18.62%. Consequently, the A + T content (55.07%) exceeded that of G + C (44.93%).

### 3.2. Haplotypes and Genetic Diversity

Genetic diversity parameters and haplotype information for all sequences are presented in Table 2 and Table 3. A total of 50 haplotypes were identified from the 122 *Cytb* sequences, based on 184 polymorphic sites. The overall genetic diversity was characterized by high haplotype diversity (*H_d_* = 0.954), substantial nucleotide diversity (*π* = 0.04765), and a mean pairwise difference (*K*) of 53.506. Similarly, the analysis of the 122 *COI* gene sequences revealed a total of 135 polymorphic sites, delineating 48 distinct haplotypes. For *COI*, the population exhibited comparably high levels of genetic diversity (*H_d_* = 0.950, *π* = 0.03251, *K* = 36.414).

### 3.3. Neutrality Tests and Mismatch Distribution Analysis

**Figure 1:**
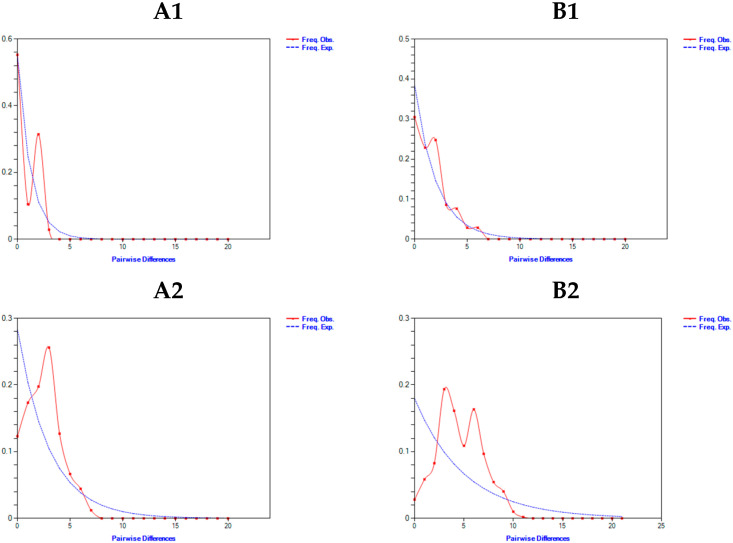

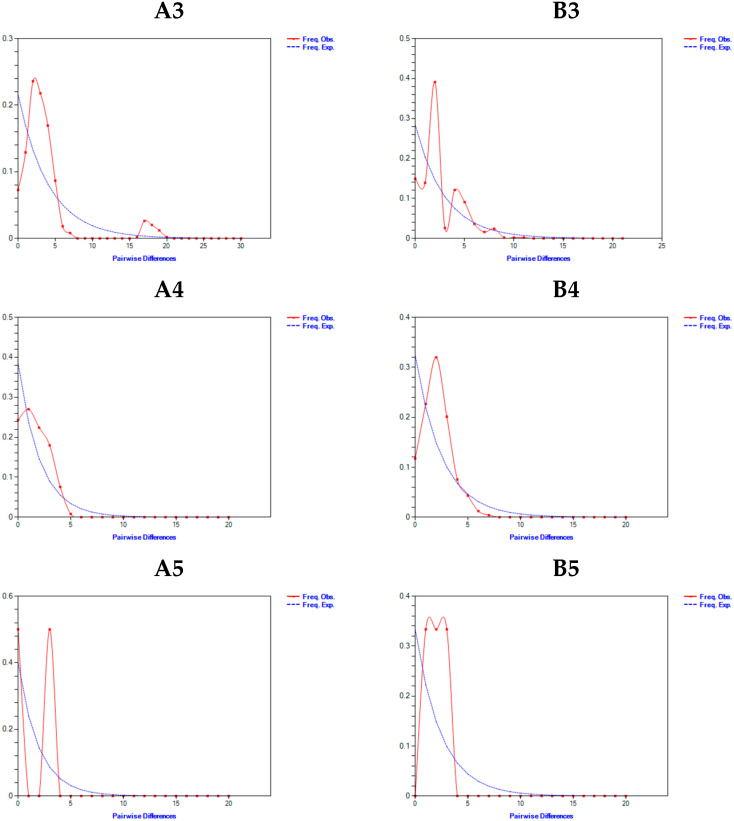
Mismatch distribution ((**A**)—*Cytb*; (**B**)—*COI*; The numbers 1–5 indicate *Cultrichthys erythropterus*, *Culter alburnus*, *Culter dabryi*, *Culter mongolicus*, and *Culter oxycephaloides*).

The results of the neutrality tests for *Cytb* gene sequences are summarized in Table 4. The *Tajima’s D* tests for *Culterichthys erythropterus*, *Culter mongolicus*, *Culter dabryi*, and *Culter oxycephaloides* all yielded negative values without statistically significant differences (*p* > 0.05). In contrast, the *Tajima’s D* test for *Culter alburnus* also indicated a negative value; however, this result was statistically significant (*p* ≤ 0.05). For *Fu’s Fs* test, both *Culterichthys erythropterus* and *Culter oxycephaloides* exhibited positive values with no statistically significant differences (*p* > 0.05). Conversely, *Culter mongolicus*, *Culter alburnus*, and *Culter dabryi* displayed negative values in *Fu’s Fs* test that were statistically significant (*p* ≤ 0.05). Nucleotide mismatch distribution analysis revealed that all species except for *Culterichthys erythropterus* had positive values for both the sum of squared deviation (*SSD*) and Harpending’s raggedness index (*Hri*), with non-significant results (*p* > 0.05). However, the mismatch distribution curves presented in Figure 1(A3,A4) indicated that only *Culter mongolicus* and *Culter dabryi* exhibited a unimodal Poisson distribution; the remaining species demonstrated multimodal mismatch distribution patterns.

The neutrality test results of the *COI* gene sequences are presented in Table 4, the *Tajima’s D* tests for *Culterichthys erythropterus*, *Culter mongolicus*, *Culter dabryi*, and *Culter oxycephaloides* all yielded negative values, but not statistically significant differences (*p* > 0.05). In contrast, the *Tajima’s D* test for *Culter alburnus* also showed a negative value, but with a statistically significant difference (*p* ≤ 0.05). For *Fu’s Fs* test, *Culter mongolicus*, *Culter alburnus*, and *Culter dabryi* all displayed negative values, and the differences were statistically significant (*p* ≤ 0.05). While *Culterichthys erythropterus* and *Culter oxycephaloides* also exhibited negative *Fu’s Fs* values, the differences were not statistically significant (*p* > 0.05). Nucleotide mismatch distribution analysis revealed that, with the exception of *Culter alburnus*, the tests for the sum of squared deviation (*SSD*) and Harpending’s raggedness index (*Hri*) were non-significant (*p* > 0.05) for all other species. However, the mismatch distribution curves (Figure 1(B3)) indicated that only *Culter dabryi* exhibited a unimodal distribution, with the remaining species showed multimodal distributions.

### 3.4. Phylogenetic Reconstruction and Haplotype Network Diagram

**Figure 2:**
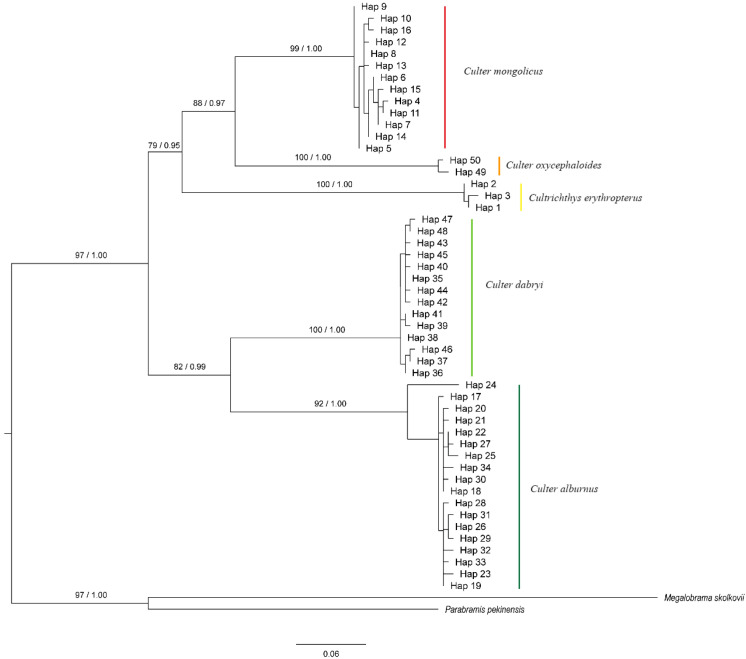
Phylogenetic tree of haplotype based on *Cytb* gene (Bootstrap value in ML and posterior probability in BI displayed at nodes).

**Figure 3:**
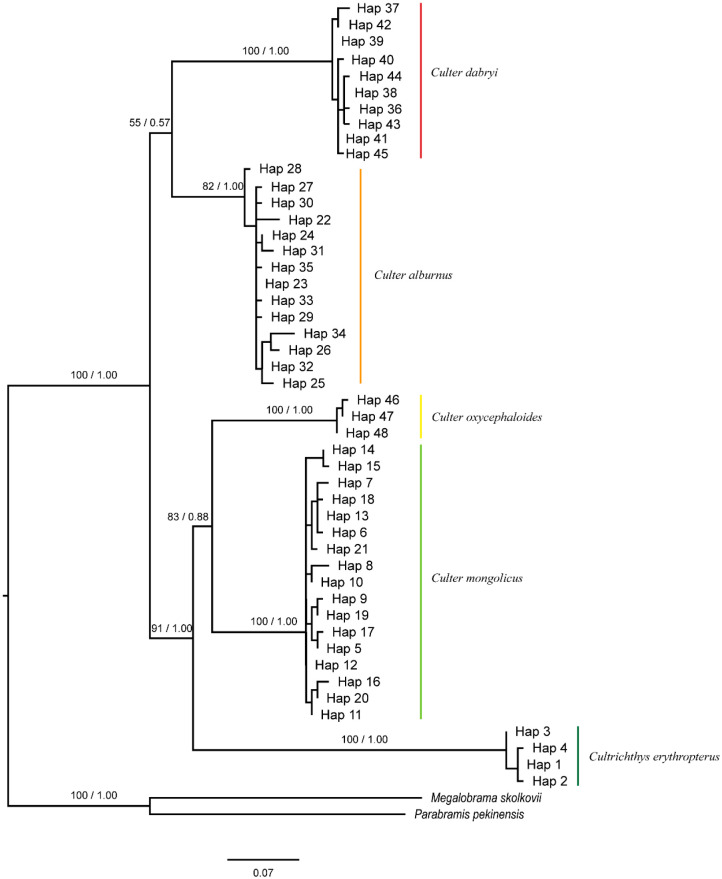
Phylogenetic tree of haplotype based on *COI* gene (BP in ML and PP in BI displayed at nodes).

**Figure 4:**
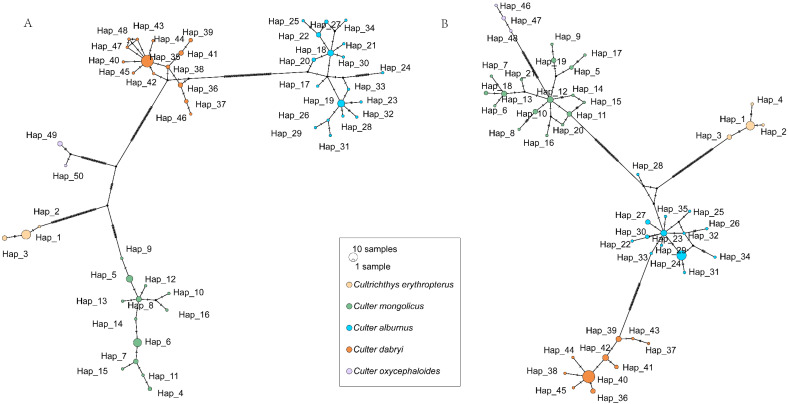
Haplotype networks of five species of culters ((**A**)—*Cytb*, (**B**)—*COI*; The size of the circle represents the number of individuals of the haplotype. Black dots represent predicted haplotypes).

In the haplotype phylogenetic trees constructed using the *Cytb* and *COI* genes (Figure 2 and Figure 3), with *Megalobrama skolkovii* and *Parabramis pekinensis* as outgroups, the genera *Culter* and *Cultrichthys* together form a strongly supported clade (*Cytb*: BP = 97%, PP = 1.00; *COI*: BP = 100%, PP = 1.00). All five Cultrinae species are resolved as distinct evolutionary lineages, each receiving robust monophyletic support. However, *Cultrichthys* is not clearly differentiated from *Culter*. Specifically, *Cultrichthys erythropterus* and *Culter oxycephaloides* are recovered as sister taxa (*Cytb*: BP = 88%, PP = 0.97; *COI*: BP = 83%, PP = 0.88). Moreover, *Culter mongolicus* is more closely related to *Cultrichthys erythropterus* and *Culter oxycephaloides*, and clusters within the same branch (*Cytb*: BP = 79%, PP = 0.95; *COI*: BP = 91%, PP = 1.00). Analysis of the *COI* sequences supports the sister-group relationship between *Culter dabryi* and *Culter alburnus* (*Cytb*: BP = 82%, PP = 0.99; *COI*: BP = 55%, PP = 0.57), corroborating their placement within the same clade. Additionally, the haplotype network based on the *Cytb* and *COI* genes (Figure 4) is largely consistent with the phylogenetic tree topology.

## 4. Discussion

### 4.1. Population Genetic Diversity

Genetic diversity serves as a key indicator of a population’s capacity to adapt to its environment. The correlation between genetic diversity and a species’ ability to adapt to environmental stress, as well as its evolutionary potential, has been extensively documented [27,28,29]. Among the commonly used metrics, haplotype diversity (*H_d_*) and nucleotide diversity (*π*) are particularly informative for assessing genetic variation [30]. Specifically, *H_d_* < 0.5 combined with *π* < 0.005 typically suggests a recent population bottleneck, whereas *H_d_* ≥ 0.5 and *π* < 0.005 indicate that a population expansion may have occurred after the bottleneck effect, resulting in an increase in haplotypes [31].

In this study, all five Cultrinae species exhibited high haplotype diversity (*H_d_* ≥ 0.5) and low nucleotide diversity (*π* < 0.005), indicating that the Cultrinae populations in Western Dongting Lake gradually recovered from the bottleneck effect but had not yet accumulated sufficient nucleotide diversity, and that the populations in such a state had a low resistance to environmental stress. It is therefore recommended that measures of conservation be taken to improve genetic diversity and maintain population stability. Consequently, it is recommended that measures be implemented to protect the population, thereby enhancing genetic diversity and maintaining population stability.

Meanwhile, this study revealed that the genetic diversity parameters based on *Cytb* and *COI* for *Culter alburnus* (*Cytb*: *H_d_*—0.927, *π*—0.00324; *COI*: *H_d_*—0.851, *π*—0.00225), *Culter mongolicus* (*Cytb*: *H_d_*—0.977, *π*—0.00225; *COI*: *H_d_*—0.972, *π*—0.00402), and *Culter dabryi* (*Cytb*: *H_d_*—0.757, *π*—0.00142; *COI*: *H_d_*—0.883, *π*—0.00186) were higher than those reported in pre-fishing ban studies by Li Daming et al. for *Culter alburnus* (*Cytb*: *H_d_*—0.907, *π*—0.0024; *COI*: *H_d_*—0.422, *π*—0.00085), *Culter mongolicus* (*Cytb*: *H_d_*—0.863, *π*—0.0024; *COI*: *H_d_*—0.695, *π*—0.00019), and *Culter dabryi* (*Cytb*: *H_d_*—0.573, *π*—0.0012; *COI*: *H_d_*—0.230, *π*—0.00053) [14,15]. This provides further evidence that the genetic diversity of these three *Culter* species has been recovering and improving since the fishing ban came into effect on 1 January 2020. However, the parameters for *Culterichthys erythropterus* (*Cytb*: *H_d_*—0.448, *π*—0.00073) were lower than those reported by Hu et al. (*Cytb*: *H_d_*—0.626, *π*—0.001) [32]. We attribute this discrepancy to the smaller sample size in the present study (15 individuals) compared to that of Hu et al. [32] (37 individuals). Additionally, this study reports for the first time the genetic diversity of *Culter oxycephaloides*, though the limited sample size also constrains the accurate assessment of genetic diversity in this species.

Regarding haplotype composition, certain haplotypes were observed to occur at notably higher frequencies. For instance, the *Culter dabrvi* haplotype was the most prevalent, accounting for 48.72% of the 14 haplotypes defined on the basis of the *Cytb* gene, and Hap40, accounting for 51.28% of the 10 haplotypes defined on the basis of the *COI* gene. This phenomenon may be attributed to the survival and subsequent adaptation of the ancestral haplotypes of this species, which persisted in a substantial number of individuals and were able to perpetuate themselves in a stable manner. Furthermore, the majority of the haplotypes of *Culter mongolicus* and *Culter alburnus* consist of only one individual. Such low-frequency haplotypes are particularly vulnerable to loss during population dynamics and evolutionary processes, potentially leading to a significant reduction in overall genetic diversity. Consequently, enhanced conservation efforts are especially warranted for these species to preserve their genetic variation and ensure long-term population resilience.

### 4.2. Population Evolutionary History

*Tajima’s D* neutrality test is a statistical comparison based on the number of segregating sites (*K*) and the average number of nucleotide differences between sequences (*π*) in a sample [33]. Between these two parameters, *K* is calculated by simply counting the number of polymorphic sites, regardless of allele frequencies; thus, even low-frequency variants exert a substantial influence on *K*. In contrast, *π*, which measures the average pairwise differences between sequences, is less affected by low-frequency variants that contribute little to average heterozygosity. Due to this differential sensitivity, when deleterious mutations arise in a population, they tend to be maintained at low frequencies due to negative selection (also known as purifying selection). This leads to an excess of low-frequency variants compared to the expectation under neutrality, resulting in a negative *D* value in DNA sequence data. Conversely, when a particular allele undergoes strong positive selection, linked neutral or slightly deleterious variants can also increase in frequency through genetic hitchhiking (or selective sweep) [34]. However, an excess of low-frequency variants may also result from the accumulation of neutral mutations [35,36,37,38,39,40]. Therefore, a negative *Tajima’s D* may indicate either the action of purifying selection or the signature of a hitchhiking event. The negative *Tajima’s D* values observed in the five Cultrinae species reveal an accumulation of low-frequency alleles within the populations, potentially implying experiences of negative selection or selective sweep. The prevalence of a substantial number of low-frequency alleles requires a sufficiently large population size as its genetic basis, indicating that all five Cultrinae species may have undergone rapid population growth in the recent past. Irrespective of the specific evolutionary mechanism, the observed excess of low-frequency alleles points to a recent rapid demographic recovery in these five species, a genetic pattern that implies a substantially large population. This recovery may be reasonably linked to the conservation effect of the ten-year fishing ban, rolled out from 1 January 2020 across the Yangtze River Basin, which eliminated all productive fishing pressure on natural stocks. However, here we need to point out that other environmental factors (e.g., habitat restoration, water quality improvement, climate change, or reduced anthropogenic disturbance beyond fishing) may also contribute to population recovery and continuous monitoring over time and in other areas of the lake would greatly strengthen this conclusion.

*Tajima’s D* and *Fu’s Fs* neutrality tests, as well as mismatch distribution analyses, have been utilised to infer the occurrence of expansion events in specific populations. Theoretically, when the results of the neutrality tests are all negative and statistically significant, and the base mismatch distribution plot has a single peak, it indicates that the population has undergone an expansion that deviates from the neutrality hypothesis. The present study examined the results of the neutrality test based on the *Cytb* gene. The results demonstrated that *Cultrichthys erythropterus*, and *Culter oxycephaloides* had accumulated a significant number of low-frequency allelic mutations within their populations. Furthermore, the populations did not expand, which was in accordance with the hypothesis of neutral evolution. The *Culter mongolicus*, *Culter dabrvi* and *Culter alburnus* had experienced significant population expansion, and the initial mutations had resulted in a deviation from the neutral evolution of the populations. The mismatch distribution map demonstrated that *Culter mongolicus* and *Culter dabrvi* had undergone population expansion, while the remaining populations exhibited stability. The results of the neutrality test based on the *COI* gene demonstrated that *Cultrichthys erythropterus* and *Culter oxycephaloides* exhibited a substantial number of low-frequency alleles within their populations and did not undergo population expansion, which was consistent with a neutral mutation. Conversely, *Culter mongolicus*, *Culter oxycephaloides* and *Culter dabrvi* deviated from a neutral mutation and underwent population expansion, as the same as *Cytb* gene. But the mismatch distribution map of *COI* gene demonstrated that *Culter dabrvi* had undergone a process of population expansion. The discrepancies observed in the outcomes of the neutrality test and mismatch analysis for the five Cultrinae species, as determined by varying genetic loci, may be ascribed to the inconsistency of the algorithms employed in disparate model analyses. The single-peaked Poisson distribution of the mismatch distribution is challenging to obtain, and the results of the sequence sequencing also influence the outcomes of the analyses. In addition, Some other reasons, such as differences in evolutionary rates between *Cytb* and *COI*, varying sensitivity of markers to demographic changes, or locus-specific selection, may also lead to the historical demographic analyses (neutrality tests and mismatch distribution) yield different results for *Cytb* and *COI*. Consequently, it can be hypothesised that the evolutionary history of the populations in real situations is more intricate.

### 4.3. Phylogeny

The phylogenetic tree illustrates the evolutionary relationships among species [41,42,43]. In this study, tree reconstruction demonstrated that the *Cytb* gene, *COI* gene, and associated mitochondrial markers effectively differentiate the five Cultrinae species examined. The genes/haplotypes of these species consistently formed monophyletic groups. Moreover, the topologies of the maximum likelihood (ML) and Bayesian inference (BI) trees, which are constructed based on the various analytical methods, were largely congruent, supporting the stability and reliability of the phylogenetic results.

Since the establishment of the genus *Culter* by Basilewsky in 1855, the ambiguity in the records and the inaccuracy of the accompanying diagrams resulted in the inclusion of *Culter* and *Erythroculter*. As a result, later researchers found it difficult to accurately distinguish between these species based solely on Basilewsky’s description [44]. Subsequent to this, Berg divided the genus *Culter* into two genera, *Culter* and *Erythroculter*, based on the character of whether the ventral keel is complete or not; subsequently, Chen et al. renamed the original *Culter* as *Protoculter* and the original *Erythroculter* as the current *Culter* [16]. Nevertheless, these genus name changes did not succeed in clarifying the confusion among the species, which led to the ongoing controversy over the classification system of *Culter* [45]. Phylogenetic analysis on Culterinae fishes based on modern molecular approaches remain relatively limited. The results of the present study demonstrated that *Culter oxycephaloides*, *Culter dabrvi* and *Culter alburnus* exhibited closer affinities, while *Culter dabrvi* and *Culter alburnus* were found to be more closely related. However, both the *Cytb* gene and the *COI* gene were unable to differentiate between the analysed genera of *Culter dabrvi* and *Culter oxycephaloides* from *Cultrichthys*. Furthermore, *Cultrichthys* was nested within *Culter oxycephaloides*, and *Culter oxycephaloides* was unable to form independent evolutionary clades, indicating the genera *Culter* and *Cultrichthys* genus were collectively recovered as a distinct clade using two molecular markers. This suggests that *Culter* is not a monophyletic group, and this is consistent with the findings of Feng et al. based on the *Cytb* gene [46], with those of Wang based on mitochondrial genome analysis [47], and with the study by Wang wei using the *COI* gene [48].

## 5. Conclusions

Genetic diversity and phylogenetic analyses provide valuable insights into species status. In this study, we examined the genetic diversity and phylogenetic relationships of five Cultrinae species in West Dongting Lake using mitochondrial *Cytb* and *COI* gene markers following the implementation of a ten-year fishing ban. The main findings are promising. High haplotype diversity (*H_d_*) coupled with low nucleotide diversity (*π*), along with negative values in neutrality tests, suggest that several species may be recovering from a past bottleneck, potentially facilitated by the fishing ban. Phylogenetically, the results corroborate earlier studies indicating that the current classification of the genus Culter may not be monophyletic—a point of relevance to systematic ichthyology. In summary, this study offers preliminary but encouraging genetic evidence that the fishing ban in West Dongting Lake could be positively affecting the recovery of key fish populations. It establishes a useful baseline for future monitoring and underscores the importance of evidence-based conservation policy.

Nevertheless, certain limitations common to studies of this kind should be noted, including small sample sizes for some species and the exclusive use of mitochondrial DNA, which primarily reflects maternal inheritance. In future work, continuous monitoring will be extended to other areas of the lake, and nuclear markers such as microsatellites or single-nucleotide polymorphisms will be employed to complement the tracking of population genetic changes.

## Figures and Tables

**Table 1:** Base composition of each mitochondrial gene sequence.

Gene	Species	Base Content/%					
		T	C	A	G	T + A	G + C
*Cytb*	*Cultrichthys erythropterus*	27.88	28.67	28.41	15.05	56.28	43.72
	*Culter mongolicus*	27.31	28.97	29.19	14.53	56.50	43.50
	*Culter alburnus*	26.73	29.46	29.29	14.52	56.02	43.98
	*Culter dabryi*	27.70	28.85	29.17	14.27	56.87	43.13
	*Culter oxycephaloides*	26.96	29.41	28.98	14.65	55.94	44.06
	Mean	27.34	29.04	29.11	14.52	56.45	43.55
*CO* *I*	*Cultrichthys erythropterus*	28.89	26.62	25.92	18.57	54.81	45.19
	*Culter mongolicus*	28.41	26.86	25.74	18.99	54.14	45.86
	*Culter alburnus*	28.88	26.19	26.49	18.44	55.37	44.63
	*Culter dabryi*	29.27	25.83	26.46	18.43	55.74	44.26
	*Culter oxycephaloides*	29.16	26.33	25.50	19.01	54.67	45.33
	Mean	28.89	26.31	26.18	18.62	55.07	44.93

**Table 2:** Genetic diversity of each mitochondrial gene.

Gene	Species	N	N_h_	S	*H_d_*	*π*	*K*
*Cytb*	*Cultrichthys erythropterus*	15	3	3	0.448	0.00073	0.819
	*Culter mongolicus*	32	13	13	0.877	0.00225	2.528
	*Culter alburnus*	32	18	33	0.927	0.00324	3.641
	*Culter dabryi*	39	14	10	0.757	0.00142	1.599
	*Culter oxycephaloides*	4	2	3	0.500	0.00134	1.500
	Total	122	50	184	0.954	0.04765	53.506
*CO* *I*	*Cultrichthys erythropterus*	15	4	8	0.695	0.00142	1.600
	*Culter mongolicus*	32	17	22	0.972	0.00406	4.573
	*Culter alburnus*	32	14	24	0.851	0.00225	2.522
	*Culter dabryi*	39	10	12	0.883	0.00186	2.089
	*Culter oxycephaloides*	4	3	4	1.000	0.00178	2.000
	Total	122	48	135	0.950	0.03251	36.414

Note: N indicates number of samples; N_h_, number of haplotypes; S, number of polymorphic sites; *H_d_*, haplotype diversity; *π*, nucleotide diversity; and *K*, mean nucleotide difference.

**Table 3:** Haplotype composition of each mitochondrial gene.

Gene	Species	Number of Haplotypes
*Cytb*	*Cultrichthys erythropterus*	Hap1(11), Hap2(1), Hap3(3)
	*Culter mongolicus*	Hap4(2), Hap5(6), Hap6(9), Hap7(3), Hap8(4), Hap9(1), Hap10(1), Hap11(1), Hap12(1), Hap13(1), Hap14(1), Hap15(1), Hap16(1)
	*Culter alburnus*	Hap17(1), Hap18(5), Hap19(7), Hap20(2), Hap21(1), Hap22(3), Hap23(1), Hap24(1), Hap25(1), Hap26(1), Hap27(2), Hap28(1), Hap29(1), Hap30(1), Hap31(1), Hap32(1), Hap33(1), Hap34(1)
	*Culter dabryi*	Hap35(19), Hap36(3), Hap37(2), Hap38(2), Hap39(2), Hap40(1), Hap41(3), Hap42(1), Hap43(1), Hap44(1), Hap45(1), Hap46(1), Hap47(1), Hap48(1)
	*Culter oxycephaloides*	Hap49(3), Hap50(1)
*CO* *I*	*Cultrichthys erythropterus*	Hap1(10), Hap2(1), Hap3(3), Hap4(1)
	*Culter mongolicus*	Hap5(2), Hap6(1), Hap7(1), Hap8(1), Hap9(1), Hap10(3), Hap11(3), Hap12(5), Hap13(4), Hap14(1), Hap15(1), Hap16(1), Hap17(1), Hap18(2), Hap19(3), Hap20(1), Hap21(1)
	*Culter alburnus*	Hap22(1), Hap23(5), Hap24(12), Hap25(1), Hap26(1), Hap27(3), Hap28(1), Hap29(1), Hap30(2), Hap31(1), Hap32(1), Hap33(1), Hap34(1), Hap35(1)
	*Culter dabryi*	Hap36(3), Hap37(1), Hap38(1), Hap39(4), Hap40(20), Hap41(2), Hap42(5), Hap43(1), Hap44(1), Hap45(1)
	*Culter oxycephaloides*	Hap46(1), Hap47(2), Hap48(1)

**Table 4:** Neutrality test and Mismatch distribution of each mitochondrial gene.

Gene		*Cultrichthys erythropterus*	*Culter mongolicus*	*Culter alburnus*	*Culter dabryi*	*Culter oxycephaloides*
*Cytb*	*Tajima’s D*	−0.33397	−0.70640	−1.99893	−0.97080	−0.75445
	*p*	0.42300	0.24200	0.00800	0.17900	0.21900
	*Fu’s Fs*	0.50999	−4.98765	−8.89613	−8.78962	1.71605
	*p*	0.54300	0.00600	0.00000	0.00000	0.74500
	*SSD*	0.06584	0.00425	0.00345	0.00161	0.25629
	*p*	0.00000	0.50000	0.19000	0.83000	0.25000
	*Hri*	0.32671	0.02853	0.02977	0.02023	0.75000
	*p*	1.00000	0.52000	0.16000	0.98000	0.57000
*CO* *I*	*Tajima’s D*	−0.99157	−0.94054	−2.02506	−1.11989	−0.70990
	*p*	0.18700	0.18700	0.00400	0.13300	0.29400
	*Fu’s Fs*	−0.15436	−7.97788	−7.00937	−4.51002	−0.88730
	*p*	0.40100	0.00000	0.00100	0.00500	0.06900
	*SSD*	0.08061	0.00074	0.02344	0.00103	0.15967
	*p*	0.18000	0.76000	0.01000	0.57000	0.24000
	*Hri*	0.33107	0.01920	0.09263	0.04326	0.75000
	*p*	0.12000	0.49000	0.00000	0.40000	0.84000

## Data Availability

The gene data of five Cultrinae fish can be obtained by contacting the author (zyshen@hunnu.edu.cn).

## References

[1] Miya M., Nishida M. (1999). Organization of the mitochondrial genome of a deep-sea fish, *Gonostoma gracile* (Teleostei: Stomiiformes): First example of transfer RNA gene rearrangements in bony fishes. Mar. Biotechnol..

[2] Liao X. (2006). Molecular Marker Screening, Development and Population Genetic Analysis of Several Important Fish Species in the Yangtze River Basin. Master’s Thesis.

[3] Sturmbauer C., Meyer A. (1992). Genetic divergence, speciation and morphological stasis in a lineage of African cichlid fishes. Nature.

[4] Subramanian S., Bonen L. (2006). Rapid evolution in sequence and length of the nuclear-located gene for mitochondrial L2 ribosomal protein in cereals. Genome.

[5] Chen G., Chang H. (1996). Research status of mitochondrial DNA polymorphism in animals. Tianjin J. Anim. Husb. Vet..

[6] Wang K., Xu Y., Cui A., Jiang Y., Wang B., Liu X., Fang L., Xue Z., Mao C. (2022). Application of DNA Barcoding Based on *Cytb*, ND1 and ND2 Genes in Species Identification of Culter Fishes. Prog. Fish Sci..

[7] Xiao W., Zhang Y. (2000). Heredity and evolution of mitochondrial DNA in fishes. Acta Hydrobiol. Sin..

[8] Guo X., Liu S., Liu Q., Liu Y. (2004). New progress in mitochondrial DNA research of fishes. Acta Genet. Sin..

[9] Qi D., Chao Y., Guo S., Zhao X. (2008). Genetic structure of mtDNA control region in five populations of *Schizopygopsis pylzovi*. Acta Zool. Sin..

[10] Yu D., Gao X., Shen Z., Fujiwara M., Yang P., Chang T., Zhang F., Wu X., Duan Z., Liu H. (2023). Novel insights into the reproductive strategies of wild Chinese sturgeon (*Acipenser sinensis*) populations based on the kinship analysis. Water Biol. Secur..

[11] Hauser L., Adcock G.J., Smith P.J., Bernal Ramírez J.H., Carvalho G.R. (2002). Loss of microsatellite diversity and low effective population size in an overexploited population of New Zealand snapper (*Pagrus auratus*). Proc. Natl. Acad. Sci. USA.

[12] Butchart S.H., Walpole M., Collen B., Van Strien A., Scharlemann J.P., Almond R.E., Baillie J.E., Bomhard B., Brown C., Bruno J. (2010). Global biodiversity: Indicators of recent declines. Science.

[13] Gao X., Fujiwara M., Winemiller K.O., Lin P., Li M., Liu H. (2019). Regime shift in fish assemblage structure in the Yangtze River following construction of the Three Gorges Dam. Sci. Rep..

[14] Li D., Liu Y., Tang K., Liu Y., Jiang Q., He H., Shen D., Zhang T. (2021). Genetic Diversity of Three *Culter* Species in the Ge Lake National Aquatic Germplasm Resources Conservation Area Based on the *COI* Gene. Jiangsu Agric. Sci..

[15] Li D., Liu Y., Tang K., Liu Y., Cai Y., Gu X., Yin J., Jiang Q., Zhang T. (2022). Genetic Diversity of Three *Culter* Species in national aquatic germplasm resource conservation area of Ge Lake based on *Cytb* gene sequence. Fish. Sci. Technol. Inf..

[16] Chen Y. (1998). Fauna Sinica, Osteichthyes, Cypriniformes (Middle Volume).

[17] Wu Y., Li H., Liao F., Yang X., Xie Z. (2021). Fish Fauna of Hunan.

[18] Xiao W., Zhang Y., Liu H. (2001). Molecular systematics of *Xenocyprinae* (Teleostei: Cyprinidae): Taxonomy, biogeography, and coevolution of a special group restricted in East Asia. Mol. Phylogenetics Evol..

[19] Cheng P. (2015). Study on Phylogenetic Pattern and Macroevolutionary Characteristics of Endemic Groups of Cyprinidae in East Asia. Ph.D. Thesis.

[20] Hall T. (1999). BioEdit: A user-friendly biological sequence alignment editor and analysis program for Windows 95/98/NT. Nucleic Acids Symposium Series.

[21] Kumar S., Stecher G., Li M., Knyaz C., Tamura K. (2018). MEGA X: Molecular evolutionary genetics analysis across computing platforms. Mol. Biol. Evol..

[22] Librado P., Rozas J. (2009). DnaSP v5: A software for comprehensive analysis of DNA polymorphism data. Bioinformatics.

[23] Bandelt H., Forster P., Röhl A. (1999). Median-joining networks for inferring intraspecific phylogenies. Mol. Biol. Evol..

[24] Excoffier L., Laval G., Schneider S. (2005). Arlequin (version 3.0): An integrated software package for population genetics data analysis. Evol. Bioinform..

[25] Stamatakis A., Aberer A., Goll C., Smith S., Berger S., Izquierdo-Carrasco F. (2012). RAxML-Light: A tool for computing terabyte phylogenies. Bioinformatics.

[26] Ronquist F., Teslenko M., Van Der Mark P., Ayres D., Darling A., Höhna S., Larget B., Liu L., Suchard M., Huelsenbeck J. (2012). MrBayes 3.2: Efficient Bayesian phylogenetic inference and model choice across a large model space. Syst. Biol..

[27] Yang H. (2021). Study on Phylogenetic Diversity of Fishes and Genetic Diversity of Four Fish Species in the Baiyangdian Watershed. Master’s Thesis.

[28] Kearney M., Spindler J., Shao W., Maldarelli F., Palmer S., Hu S., Lifson J., KewalRamani V., Mellors J., Coffin J. (2011). Genetic diversity of simian immunodeficiency virus encoding HIV-I reverse transcriptase persists in macaques despite antiretroviral therapy. J. Virol..

[29] Hughes A., Inouye B., Johnson M., Underwood N., Vellend M. (2008). Ecological consequences of genetic diversity. Ecol. Lett..

[30] Bonin A., Nicole F., Pompanon F., Miaud C., Taberlet P. (2007). Population adaptive index: A new method to help measure intraspecific genetic diversity and prioritize populations for conservation. Conserv. Biol..

[31] Grant W., Bowen B. (1998). Shallow population histories in deep evolutionary lineages of marine fishes: Insights from sardines and anchovies and lessons for conservation. J. Hered..

[32] Hu Y., Yang S., Li M., Cao W., Liu H. (2015). Population differentiation of *Cultrichthys erythropterus* in Poyang Lake and Dongting Lake. Acta Hydrobiol. Sin..

[33] Tajima F. (1983). Evolutionary relationship of DNA sequences in the infinite populations. Genetics.

[34] Tajima F. (1989). Statistical method for testing the neutral mutation hypothesis by DNA polymorphism. Genetics.

[35] Kimura M. (1968). Evolutionary rate at the molecular level. Nature.

[36] Kimura M. (1983). The Neutral Theory of Molecular Evolution.

[37] Zhou Q., Wang W. (2004). Detecting Natural Selection at the DNA Level. Zool. Res..

[38] Fay J., Wyckoff G., Wu C. (2002). Testing the neutral theory of genomic data from *Drosophila*. Nature.

[39] Fu Y. (1997). Statistical tests of neutrality of mutations against population growth, hitchhiking and background selection. Genetics.

[40] Fu Y., Li W. (1993). Statistical tests of neutrality of mutations. Genetics.

[41] Bernardi G. (2020). Isochores and the evolutionary genomics of vertebrates. Gene.

[42] Avise J. (2006). Evolutionary Pathways in Nature: A Phylogenetic Approach.

[43] Wang B. (2022). Phylogenetic Tree Construction and Evaluation Method Based on Dynamic Multi-Objective Optimization. Master’s Thesis.

[44] Wu X., Cao W., Yi B., Yang G., Huang H., Wu Q. (1964). Fauna Sinica, Cyprinidae (Upper Volume).

[45] Yi B., Zhu Z. (1959). A study on *Culter* and *Erythroculter* fishes in China. Collect. Pap. Hydrobiol..

[46] Feng X., Xie N., Feng J., Xu B., Zhao J. (2009). Phylogenetic relationships of the Culterinae fishes based on cytochromeb sequences analysis. Freshw. Fish..

[47] Wang J. (2013). Molecular Phylogenetic Relationships of Cultrinae Sensu Stricto and Related Groups (Teleostei: Cyprinidae) and Genetic Diversity of Megalobrama Pellegrini (Cultrinae). Master’s Thesis.

[48] Wang W., Chen L., Yu N., Jiang Z. (2008). Genetic diversity analysis of *Culter alburnus* populations based on partial sequence of *COII* gene. Dalian Fish. Univ. J..

